# Panton-Valentine Leukocidin (PVL) genes may not be a reliable marker for community-acquired MRSA in the Dakahlia Governorate, Egypt

**DOI:** 10.1186/s12866-023-03065-8

**Published:** 2023-10-28

**Authors:** Mohamed Shohayeb, Tarek El-Banna, Lamis E. Elsawy, Maisra M. El-Bouseary

**Affiliations:** 1 Department of Microbiology and Immunology, Faculty of pharmacy, Delta University for Science and Technology, Gamasa, Egypt; 2 Department of Pharmaceutical Microbiology, Faculty of Pharmacy, Tanta University, Tanta, Egypt

**Keywords:** MRSA, Panton-Valentine leukocidin (*PVL*), *mecA*, SCC*mec* types, MDR, Egypt

## Abstract

**Background:**

Methicillin-resistant *Staphylococcus aureus* is linked to both nosocomial and community infections. One of the key virulence factors of *S. aureus* is Panton-Valentine leukocidin (*PVL*). The *PVL* genes are mostly associated with community-acquired MRSA (CA-MRSA). This study evaluates the prevalence of *PVL* genes as a marker for CA-MRSA at tertiary hospitals in Mansoura, Dakahlia, Egypt. *S. aureus* was isolated from clinical specimens obtained from different departments of tertiary hospitals, outpatient clinics, and hospital healthcare workers (HCWs). PCR was used to detect the *mecA*, *PVL*, and SCC*mec* genes among the recovered isolates. Standard broth microdilution method was used to determine the minimum inhibitory concentrations (MIC) of nine antibiotics against *S. aureus*.

**Results:**

Two hundred *S. aureus* isolates were recovered and identified out of the total isolates (n = 320). The *mecA* gene was detected in 103 *S. aureus* isolates (51.5%). Among the MRSA isolates, 46.60% were *PVL*-positive. The incidence of the *PVL* genes of MRSA in nosocomial (HA), outpatient clinics (CA), and HCWs was 46.66%, 56.52%, and 42%, respectively. All MRSA isolates showed resistance to cefoxitin. The percentage of resistance to most tested antibiotics was high, except for ciprofloxacin (6.85%). Both antibiotic resistance and multidrug resistance among MRSA isolates were generally higher in *PVL-*positive isolates than in *PVL-*negative isolates in HA- and CA-MRSA isolates. While SCC*mec* type V was the most prevalent in *PVL*-positive MRSA stains, type I was the most prevalent in *PVL*-negative isolates.

**Conclusion:**

This study revealed that *PVL* genes are generally highly prevalent among *mecA-*positive MRSA isolates, whether they are CA-MRSA, HA-MRSA, or HCW isolates. Therefore, *PVL* is not a valid marker for CA-MRSA in Mansoura, Dakahlia Governorate, Egypt, as has been reported in other countries. Further epidemiologic studies are required to track the incidence of *PVL* in HA-MRSA, CA-MRSA, and HCW isolates in other Egyptian governorates.

## Introduction

*Staphylococcus aureus* is the most aggressive species of staphylococci and causes a wide range of symptoms, from skin and soft tissue infections to potentially lethal pneumonia and toxin-related illnesses like toxic shock syndrome. It is also one of the most frequently isolated pathogens from hospitalised patients and the second-most frequently isolated pathogen from people in outpatient settings [1]. Although the discovery of penicillin in the 1940s helped to reduce the high mortality rate caused by *S. aureus*, this was short-lived because resistant isolates producing β-lactamases quickly evolved, and most *S. aureus* infections in hospitals became penicillin-resistant [2]. In the late 1950s, methicillin, which is a β-lactam antibiotic insensitive to beta-lactamases, offered a new option for treating resistant *S. aureus* infections. However, soon, methicillin-resistant *S. aureus* (MRSA) isolates appeared primarily in healthcare settings. MRSA isolates are resistant to all β-lactams, including penicillins and cephalosporins [2]. In addition to β-lactam resistance, MRSA isolates gradually developed resistance to other antibiotic classes [3]. Currently, multidrug resistance (MDR) is highly prevalent in MRSA isolates, and it is one of the most important current threats to public health [4].

MRSA resistance to β-lactam antibiotics is linked to the *mecA* gene, which encodes penicillin-binding protein 2a (PBP2a). PBP2a has a poor affinity for all β-lactams and maintains the transpeptidase activity in MRSA isolates in the presence of lethal amounts of β-lactams [5]. PBP2a is encoded by the *mecA* gene, which is contained in a movable genetic component known as the Staphylococcal chromosome cassette *mec* (SCC*mec*) [5, 6]. *mecC* MRSA is a recently identified subtype of MRSA that encodes a divergent *mec* gene and may colonise and infect a broad variety of host species, including humans. Although *mecC* MRSA isolates are currently uncommon and have been identified mainly in Europe, they could pose a diagnostic challenge when *mecA* or PBP2a/2′ detection is only used to diagnose MRSA [7]. Alongside their rise in nosocomial infections, MRSA isolates from community patients have become globally prevalent, and reports of serious and rapidly progressive fatal disease due to virulent community-acquired MRSA (CA-MRSA) have currently alarmed healthcare professionals [8].

*S. aureus* has a variety of virulence factors that enable it to evade the immune system and attach to and injure the host. These factors are related to the pathogenicity of the bacterium. One virulence factor of *S. aureus* that has a particular importance is Panton–Valentine leukocidin (*PVL*). It is composed of two separate proteins encoded by two adjacent genes, *lukS-PV and lukF-PV* [9]. The bicomponent toxin is a pore-forming leukotoxin that lyses leukocytes and has been initially associated with severe skin and soft tissue infections and necrotizing pneumonia [10].

The *PVL* genes were initially and frequently more associated with CA-MRSA isolates and were rarely found in hospital-acquired MRSA (HA-MRSA) isolates. However, subsequently, they were also detected among HA-MRSA isolates [11]. Therefore, in this study, we investigated the prevalence of *PVL* genes among HA-MRSA, CA-MRSA, and HCWs collected from Mansoura tertiary hospitals, Dakahlia Governorate, Egypt.

## Materials and methods

### **Specimens’ collection**

Non-duplicated specimens of pus, urine, blood cultures and nasal nares swabs were screened for *S. aureus* from April 2020 to February 2021. The specimens were collected from different hospital departments, at Mansoura University tertiary hospital labs that serve Dakahlia citizens. The specimens were obtained from 69 inpatients, 50 outpatient clinics and 81 HCWs. They were collected from eyes, pus, blood and urinary tract infections. Hospital-acquired *S. aureus* isolates were obtained from patients after admission to tertiary hospitals for more than 72 h. Community-acquired isolates were obtained from outpatient departments for patients who had not previously been hospitalised within the last 6 months. Nasal swaps were obtained from health care workers, including doctors, nurses, laboratory technicians, and housekeeping staff.

### MRSA isolation and identification

Isolates were identified as *S. aureus* by standard methods. They were plated out on several culture media, including blood agar (Oxoid™, Thermo Scientific, USA), mannitol salt agar (Oxoid™, Thermo Scientific, USA), and nutrient agar (Oxoid™, Thermo Scientific, USA). Gram-staining, catalase and coagulase assays were used to confirm morphologically suspicious staphylococcal colonies [12]. The identified *S. aureus* isolates were kept at -80 °C in a tryptone-soy broth (Oxoid™, Thermo Scientific, USA) containing 20% glycerol, for subsequent testing. To primarily detect MRSA, *S. aureus* isolates were screened by disc diffusion susceptibility tests using 30 µg cefoxitin discs (Oxoid™, Thermo Scientific, USA) and Mueller-Hinton agar (Oxoid™, Thermo Scientific, USA) according to the CLSI guidelines [13].

### Antibiotic susceptibility testing (AST)

Antibiotic susceptibility testing was performed by standardised broth microdilution for the determination of the minimal inhibitory concentration (MIC), according to the CLSI guidelines [13]. Briefly, bacterial turbidity was adjusted to 0.5 McFarland standard. Antibiotics were twofold serially diluted in Mueller-Hinton broth in microtiter plates. Wells were inoculated with bacterial suspensions in Muller-Hinton broth and incubated for 18 h. The MIC was calculated as the lowest concentration of the antibiotic that completely inhibited the growth of the tested organism in microtiter wells [13].

### **Screening isolates for*mecA***, ***PVL*and SCC*mec*types (**I-V) **genes by polymerase chain reaction (PCR)**

DNA was extracted from the MRSA isolates by boiling method [14]. The primers used for *mecA*, *PVL* and SCC*mec* genes (QIAGEN, USA), are listed in Table 1. For all genes, the thermocycler (FPROGO2D, Techni LTD, Oxford Cambridge, UK) was programmed for an initial denaturation at 94 °C for 4 min, followed by 30 amplification cycles and a final extension at 72 °C for 2 min. For *mecA*, the cycles were denaturation at 94 °C for 30s, annealing at 55 °C for 30s, and extension at 72 °C. For *PVL* the amplification cycle was denaturation at 94 °C for 45s, annealing at 56 °C for 45s and extension at 72 °C for 30s.

**Table 1:** Primers for detection of *mecA* , *PVL*, and SCC*mec*.

Gene	Type	Nucleotide Sequence (5’ to3’)	Amplicone size (bp)	Reference
***mecA***	Fw	AAA ATC GAT GGT AAA GGT TGG C	585	[49]
	Rv	AGT TCT GCA GTA CCG GAT TTG C		
***PVL***	Fw	ATC ATT AGG TAA AAT GTC TGG ACA TGA TCC A	433	[51]
	Rv	GCA TCA AGT GTA TTG GAT AGC AAA AGC		
**SCC** ***mec***	β	ATTGCCTTGATAATAGCCYTCT	937	[50]
	α3	TAAAGGCATCAATGCACAAACACT		
	ccrCF	CGTCTATTACAAGATGTTAAGGATAAT	518	
	ccrCR	CCTTTATAGACTGGATTATTCAAAATAT		
	1272F1	GCCACTCATAACATATGGAA	415	
	1272R1	CATCCGAGTGAAACCCAAA		
	5RmecA	TATACCAAACCCGACAACTAC	359	
	5R431	CGGCTACAGTGATAACATCC		

For SCC*mec* the cycles were denaturation at 94 °C for 30 s, annealing at 55 °C for 30s and extension at 72 °C for 60s. Seven µl of PCR products were analysed by electrophoresis at 100 V for 45 min on 1.5% agarose gel containing ethidium bromide (Sigma-Aldrich®^,^ Germany) followed by visualisation using a UV transilluminator (Hoefer™, USA) at 312 nm.

### Statistical analysis

The Chi-square test was used for the statistical evaluation of different parameters between two groups using SPSS, ver. 17. *P*-value of ≤ 0.05 was considered statistically significant.

## Result

### Isolation and identification of MRSA

A total of 200 *S. aureus* were isolated from different hospital departments, outpatient clinics, and nasal swabs of health care workers. The prevalence of MRSA among *HCWs*, outpatients, and inpatients was 43.5% (50/81), 46% (23/50), and 61.7% (30/69), respectively. *As shown in* Table 2, *the PVL* genes were relatively more prevalent in pus samples (64.28%), followed by blood (60.00%). The overall prevalence of the *PVL* genes among MRSA was 46.60% (48/103), distributed as follows: 46.60% HA-MRSA, 56.52% CA-MRSA, and 42% HCWs (Table 3).

**Table 2:** Distribution of *PVL* genes in different MRSA clinical isolates according to the clinical specimen source

Clinical Specimen Source	*PVL* positive (%)
**Eye**	5/14 (35.71%)
**Pus**	9/14 (64.28%)
**Urine**	4/10 (40.00%)
**Blood**	9/15 (60.00%)
**Health care workers**	21/50 (42.00%)
**Overall incidence**	48/103 (46.60%)

**Table 3:** Incidence of MRSA and *PVL* genes in in-patients, out-patients and health care workers

Isolated from	Incidence of MRSA	*PVL*-positive
**In-patients clinics**	43.5% (30/69)	46.67% (14/30)
**Out-patients**	46% (23/50)	56.52% (13/23)
**Health Care Workers**	61.7% (50/81)	42.00% (21/50)

### Antibiotic susceptibility of MRSA

Cefoxitin resistance was demonstrated in all MRSA isolates (Table 4**)**. A relatively high percentage of resistance was observed for *PVL*-positive than *PVL-*negative isolates. Resistances to clindamycin and gentamicin were statistically higher in *PVL*-positive than *PVL-*negative isolates (*P < 0.05*). All recovered MRSA isolates (n = 103) were multidrug-resistant (MDR). Out of the nine classes of tested antibiotics, *PVL*-negative isolates were resistant to 3–8 classes and *PVL*-positive isolates were resistant to 5–8 antibiotics classes (Table 5).

**Table 4:** Antibiotic resistance in *PVL* positive and *PVL* negative isolates

Antibiotic Class	Antibiotic Name	*PVL*-positive		*PVL*-negative		*Total % of Resistance*
		*MIC range (µgml* ^*− 1*^ *)*	*% of Resistance*	*MIC range (µgml* ^*− 1*^ *)*	*% of Resistance*	
β-lactams	Cefoxitine	8->256	100%	8->256	100%	100.00%
Macrolides	Erythromycin	4->256	97.9%	1->256	94.5%	96.20%
Quinolones	Ciprofloxacin	< 0.25-8	8.3%	< 0.25-16	5.4%	6.85%
Aminoglycosides	Gentamicin*	< 0.25->256	87.5%	1->256	67.2%	83.75%
Tetracyclines	Tetracycline	0.5->256	87.5%	0.5->256	80%	83.75%
lincomycin	Clindamycin*	0.25–128	83.3%	0.25–128	58.2%	70.75%
Glycopeptide	Vancomycin	< 0.25->256	25%	< 0.25->256	21.8%	23.4%
Folic acid inhibitors	Co-trimoxazole	0.5->256	64.6%	0.5->256	50.9%	57.75%
Fusidane	Fusidic acid	4->256	97.9%	4->256	96.3%	97.1%

* Clindamycin and gentamicin resistances were significantly higher in *PVL*-positive than *PVL-*negative isolates (*P < 0.05*)

**Table 5:** Resistance patterns of *PVL*-MRSA positive and negative strains in hospital acquired (HA), community acquired (CA) and health care workers (HCW).

Code	Number of Antibiotics	Pattern*	Frequency in						
			HA		CA			HCW	
			*PVL+*	*PVL-*		*PVL+*	*PVL-*	*PVL+*	*PVL-*
**1**	2	Cef, Ere		1					
**2**	3	Cef, Ere, Fus					1		
**3**	3	Cef, Gen, TS							1
**4**	4	Cef, Ere, Tet, Fus		1					2
**5**	4	Cef, Ere, Tet, Van		1					
**6**	4	Cef, Ere, Gen, Fus					1		
**7**	4	Cef, Gen, Tet, Fus							1
**8**	5	Cef, Ere, Gen, Van, Fus		1					
**9**	5	Cef, Ere, Gen, Cli, Fus		1			1	1	
**10**	5	Cef, Ere, Tet, TS, Fus		1		1			
**11**	5	Cef, Ere, Gen, Tet, Fus				2	1	2	
**12**	5	Cef, Ere, Tet, Cli, Fus				2	1		2
**13**	5	Cef, Tet, Van, TS, Fus							1
**14**	5	Cef, Ere,, Cli, TS, Fus							1
**15**	5	Cef, Ere, Gen, TS, Fus							1
**16**	6	Cef, Ere, Gen, Tet, TS, Fus						1	3
**17**	6	Cef, Ere, Cip, Tet, Cli, Fus		1					
**18**	6	Cef, Ere, Gen, Cli, TS, Fus	1	1				1	1
**19**	6	Cef, Ere, Tet, Cli, Van, Fus		1					
**20**	6	Cef, Ere, Gen, Tet, Cli, Fus	3	1		2	2	4	4
**21**	6	Cef, Ere, Tet, Van,TS, Fus					1		1
**22**	6	Cef, Ere, Tet, Cli, TS, Fus				1			1
**23**	6	Cef, Ere, Gen, Tet, TS, Cli	1						
**24**	6	Cef, Gen, Tet, Cli, TS, Fus	1						
**25**	7	Cef, Ere, Tet, Cli, Van, TS, Fus	1					1	1
**26**	7	Cef, Ere, Gen, Tet, Va, TS, Fus		1				2	1
**27**	7	Cef, Ere, Gen, Tet, Cli, TS, Fus	2	3		3	2	5	4
**28**	7	Cef, Ere, Gen, Tet, Cli, Van, Fus		1					
**29**	7	Cef, Ere, Cip, Gen, Tet, Cli, Fus						1	1
**30**	7	Cef, Ere, Gen, Cli, Van, TS, Fus						1	1
**31**	7	Cef, Ere, Cip, Gen, Cli, TS, Fus	1					1	
**32**	8	Cef, Ere, Gen, Tet, Cli, Van, TS, Fus	3	1		2		1	
**33**	8	Cef, Ere, Cip, Gen, Tet, Cli, TS, Fus							2
**34**	8	Cef, Ere, Cip, Gen, Tet, Cli, Van, Fus	1						
		Total	14	16		13	10	21	29

* Cef: Cefoxitine, Ere: Erythromycin, Cip: Ciprofloxacin, Gen: Gentamicin, Tet: Tetracycline, Cli: Clindamycin, Van: Vancomycin, TS: Co-trimoxazole, Fus: Fusidic acid

Most MRSA isolates (63.1%) were resistant to six to seven antibiotic classes. While the percentages of resistance to seven antibiotics in *PVL*-positive in HA, CA and HCWs isolates were 71.4%, 46.0% and 81.0%, respectively. In *PVL*-negative isolates, their percentages were 62.51%, 40.0%, and 62.7%, respectively (Table 5).

The most commonly observed resistance pattern in MRSA isolates was: Cef, Ere, Gen, Tet, Cli, TS, Fus. This pattern was detected in 18.44% of the MRSA isolates.

### Detection of *mecA*, *PVL* and SCC*mec* genes

MRSA-positive isolates were screened for SCC*mec* types. The most common type among the *PVL*-positive isolates was type V. However, type I was the most prevalent among the *PVL*-negative isolates. The rate of type V SCC*mec* gene was significantly higher (*p ≤ 0.05*) in *PVL*-positive compared to *PVL-*negative. Type IV SCC*mec* gene was detected in 22.92% and 23.62% of *PVL*-positive and *PVL*-negative isolates, respectively. Type II SCC*mec* was not detected in any of the *PVL*-positive or negative isolates. The other unidentified SCC*mec* types were 22.92% and 27.26% in *PVL*-positive and *PVL-*negative isolates, respectively (Table 6).

**Table 6:** Types of SCC*mec* distributed in *PVL* positive and *PVL* negative isolates

SCCmec*A* Type	*PVL*-positive				*PVL*-negative			
	CA	HA	HCW	Total (%)	CA	HA	HCW	Total (%)
I^*^	2		2	4 (8.33%)	4	2	10	16 (29.09%)
II	-	-	-	-	-	-	-	-
III	-	-	2	2 (4.16%)	1	-	2	3 (5.44%)
IV	4	1	6	11 (22.92%)	3	3	7	13 (23.62%)
V^*^	3	8	9	20 (41.67%)	-	3	5	8 (14.53%)
Others	4	5	2	11 (22.92%)	2	8	5	15 (27.26%)
Total	13	14	21	48 isolates	10	16	29	55 isolates

CA: Community acquired, HA: Hospital acquired, HCW: Health care workers

^*^ The rate of type V SCC*mec* was significantly higher (*P ≤ 0.05*) in *PVL*-positive compared to *PVL-*negative isolates

## Discussion

The widespread proliferation of MRSA is a major public health issue that challenges clinicians all over the world [15]. MRSA first emerged in the 1960s after being isolated from clinical specimens of hospitalised patients. Since the early nineties, MRSA has expanded quickly in communities all over the world. However, a decrease in the rates of MRSA infections has been reported in the UK and several developed countries [16]. On the contrary, the literature suggests an increase in MRSA infections in developing countries [17, 18]. However, there is a lack of a national surveillance system in Egypt to extrapolate the data obtained in different cities to have nationwide data. Reports described a general MRSA prevalence rate of 50–82% among patients hospitalised in two large Egyptian cities, Cairo and Alexandria, and a lower rate of 24% in the less populated City of Minia in southern Egypt [19]. The prevalence of CA-MRSA in Egypt ranges between 19 and 47% [20].

In this study, the overall incidence of MRSA was 51.5% (103/200), which is very high compared to the USA and Europe [23–25]. In addition, its incidence was relatively higher among HCWs (61.7%) compared to CA-MRSA (46%) and HA isolates (43.5%). The pathogenicity of *S. aureus* and its resistance to antibiotics are detrimental factors in the treatment of infections [21]. One of the key virulence factors of *S. aureus* is *PVL*, which increases the pathogenicity of *S. aureus* by accelerating apoptosis and destroying polymorphonuclear and mononuclear cells [10]. Generally speaking, the prevalence of *PVL* genes among MRSA isolates has been increasing in different countries [22–24]. However, it differs from one country to another [25, 26]. European countries with low overall rates of *PVL*-positive MRSA isolates include the United Kingdom (0.06%) [27], Germany (6.2%) [28], Ireland (1.8%) [29] and the Netherlands (15%) [30]. Low incidences were also reported in some Asian countries, like China (12.8%) [31] and Japan [11]. On the contrary, relatively higher rates of *PVL*-positive MRSA are common in Mediterranean countries like Turkey (18.3%) [32] and France (33.8%) [33].

In Egypt, there are a few studies on the molecular epidemiology of *PVL*-encoding MRSA isolates [34–36]. In the present study, the overall rate of detection of *PVL* genes was 46.60% (48/103) among MRSA isolates. This rate of prevalence of the *PVL* genes is higher than the rates in European, Asian, and Mediterranean countries. Previous reports from three different geographical locations in Egypt suggested different rates of prevalence of *PVL* genes. PVL-MRSA prevalence rates in Cairo and Ismailia were 19.04% and 92.2% in CA-MRSA, respectively, and 22.2% and 28.6% in HA-MRSA [34, 35]. On the other hand, the incidence of *PVL* genes was 16.67% in HCWs in the city of Fayoum [36]. In our study, a generally high incidence of *PVL*-positive MRSA in HA-MRSA, CA-MRSA, and HCWs was detected. The *PVL*-MRSA prevalence was 42.00%, 46.60%, and 56.52% in HA, CA, and HCWs, respectively.

The strikingly higher incidence of MRSA and *PVL*-MRSA in HA, CA, and HCWs in this study may be attributed to factors such as the excessive use of antibiotics due to the availability of antibiotics without prescription, the prescription of antibiotics for viral infections, as well as the absence of rapid and accurate methods for identification and decolonization of carries. On the other hand, one of the principal infection control measures for limiting the spread of nosocomial MRSA infection involves the performance of admission screening cultures for MRSA and the isolation of colonized or infected patients [37]. This protocol does not seem to be strictly implemented in tertiary hospitals of Mansoura. In addition, in tertiary hospitals in Egypt, there is a patient escort system. In this case, a person close to the patient stays with him in his room. So, the patient companions should also be screened for MRSA, otherwise, they contribute to the spread of CA-MRSA in hospitals. Therefore, it is not surprising that the incidence of MRSA isolates recovered from HCWs was 61.7%. and the incidence of *PVL*-positive isolates was 42%.

The high carriage rate of *PVL*-MRSA isolates in HCWs (42.0%) detected in this study suggests that they act as a reservoir for *PVL-*MRSA isolates and shed these isolates in both tertiary hospitals and the community. Therefore, strict control measures for the carriage of MRSA by patients admitted to hospitals and health care workers should be implemented.

Concerning antibiotic susceptibility testing, all recovered MRSA isolates (n = 103) were multidrug resistant, according to the European Centre for Disease Prevention and Control (ECDC) and the Centres for Disease Control and Prevention (CDC) [38]. All MRSA isolates were cefoxitin-resistant. The high levels of resistance rates for erythromycin, gentamicin, tetracycline, and clindamycin were observed (97.9%, 87.5%, 87.5% and 83.3%, respectively). This may be attributed to class I integrons, which are common in MRSA isolates from Egypt [39]. Class I integron has been associated with the resistance of staphylococci to several antibiotics, including erythromycin, gentamicin, tetracycline, and trimethoprim/sulfamethoxazole [40].

A lower overall resistance rate of 6.85% was observed for ciprofloxacin. This confirms a previous report of a low resistance rate for CA-MRSA in Mansoura [41]. It seems that ciprofloxacin is not very commonly prescribed at Mansoura, and therefore, MRSA isolates are still highly sensitive to it. In contrast, 96% of the MRSA isolates at Alexandria Main University Hospital are resistant to ciprofloxacin [42]. The overall rate of resistance to vancomycin in this study was 23.4%. Therefore, vancomycin can still be used for the treatment of MRSA isolates and should be guided by culture sensitivity testing. Ibrahiem et al. reported that among 127 MRSA isolates that were recovered from 268 clinical samples collected from different locations in Egypt, the percentage of vancomycin-resistant isolates was 23.62% [43]. Another study conducted in Egypt revealed that the prevalence of VRSA was 21.7% [44]. The former observations indicate an alarming increase in vancomycin resistance in different areas of Egypt that could be attributed to the overuse of vancomycin for treating MRSA infections. As a result, there is an urgent need to establish adequate surveillance for antibiotic resistance and implement a restricted antibiotic policy in Egyptian hospitals.

We screened the tested MRSA isolates for their SCC*mec* types (I–V) in the *mecA*-positive isolates. SCC*mec* types I, II, and/or III were reported in HA-MRSA isolates [45], whereas SCC*mec*-variant genes IV and V were detected in CA-MRSA isolates [46–48]. In the present study, type V SCC*mec* was the most prevalent among *PVL-positive* MRSA isolates (41.67%), compared to *PVL*-negative isolates (14.53%). The SCC*mec* gene variant type IV, which was commonly reported in CA-MRSA [49], was equally associated with *PVL*-positive (22.92%) and *PVL*-negative (23.62%) MRSA isolates. The most prevalent SCC*mec* type among the *PVL*-negative isolates was type I, as previously reported [50], and its incidence rate in our study is 29.09%. On the contrary, type II was not detected in any of the *PVL*-positive or negative isolates. *PVL* genes have been previously reported as markers for CA-MRSA [50].

In the current study, the carriage of MRSA isolates by HCWs was 61.7%, reflecting their major role in spreading MRSA in tertiary hospitals and the community of Dakahlia Governorate. Therefore, there is a need for rapid identification of MRSA and appropriate infection control measures for MRSA infection. Also, improving compliance, reducing antibiotic overuse, screening HCWs for carriage of *S. aureus*, and decolonization are necessary strategies. The high incidence of *PVL-*positive MRSA suggests that it is not a good marker for community MRSA in Egypt.

## Conclusions and future perspectives

In conclusion, there is an alarmingly high prevalence of MDR in *PVL-*positive and negative MRSA in tertiary hospitals and in the community in Mansoura, Dakahlia Governorate. This necessitates the reduction of the excessive use of antibiotics and the implementation of MRSA control measures in tertiary hospitals in Dakahlia Governorate. The high incidence of *PVL*-MRSA in HA, CA, and HCWs in this study suggests that *PVL* is not a valid marker for CA-MRSA in Dakahlia Governorate, Egypt. Further epidemiologic studies using different molecular typing techniques should be conducted to identify the circulating MRSA clone complexes (CCs), and their sequence type and relate the isolates to their origin, whether CA or HA in Dakahlia Governorate, Egypt.

## Data Availability

All data generated or analysed during this study are included in this published article.
